# Improved first arrival picking of microseismic P-waves in coal mines using multi-denoising and adaptive characteristic functions

**DOI:** 10.1038/s41598-025-18503-y

**Published:** 2025-09-26

**Authors:** Yong Zhang, Kai Zhan, Ping Song, Chuntao Liang, Rui Xu, Chao Kong

**Affiliations:** 1 Yankuang Energy Group Company Limited, Jining, 273500 China; 2 Chengdu University of Technology, Chengdu, 610059 China; 3 Shandong Keyue Technology Company Limited, Jinan, 250014 China

**Keywords:** IWTSE-MSDACF-AIC, Improved complete ensemble empirical mode decomposition with adaptive noise, Sample entropy, Wavelet threshold denoising, P-wave first arrival picking, Geophysics, Energy science and technology, Engineering

## Abstract

Microseismic monitoring is a critical technology for mine safety monitoring, but existing microseismic picking methods exhibit instability and limitations when dealing with high-noise data in coal mine environments. This paper proposes a method for improving the picking of high-noise microseismic P-wave first arrivals, called IWTSE-MSDACF-AIC. The method first uses the improved complete ensemble empirical mode decomposition with adaptive noise to decompose the microseismic signal into a series of intrinsic mode functions (IMFs). Then, the sample entropy of the IMFs is calculated, and an appropriate threshold is set to perform wavelet denoising on the IMFs. The signals are then reconstructed to distinguish noise from useful signals. Finally, the denoised signal’s P-wave first arrival is automatically determined using the proposed picking method based on the moving standard deviation-adaptive characteristic function and Akaike information criterion, which incorporates the relative energy coefficient and relative energy time series. Tests using synthetic seismic records with different signal-to-noise ratios and validation on real coal mine seismic datasets show that the proposed denoising strategy and picking method achieve high accuracy and robustness. In practical data tests, 90.01% of data errors fell within the range of 0s to 0.06s, demonstrating excellent picking performance. Furthermore, in grid search localization using five calibration blasts at the Dongtan coal mine, the localization results based on the proposed method significantly outperformed those based on traditional methods and PhaseNet.

## Introduction

Microseismic monitoring technology, as a reliable and effective tool, has been widely applied in various fields of mine safety, including stress monitoring of surrounding rock masses^1^ hydraulic fracturing monitoring^2^ and identification of hazardous slope areas^3^. The accurate picking of microseismic arrivals is the primary task and key step in signal analysis. The precision of microseismic arrival picking directly determines the accuracy of event identification, localization, velocity tomography, and source mechanism solutions.Currently, several well-established methods are used to evaluate the arrival times of high-quality microseismic records, including the Short-Term Average/Long-Term Average (STA/LTA) method^4,5^ Modified Akaike Information Criterion (M-AIC) methods^6^ the Kurtosis method^7^ and deep learning approaches^8–11^. In addition, various methods have been developed to suppress noise in noisy microseismic records, laying the foundation for combined picking approaches. For instance, by detecting the polarization characteristics of the signal, polarization filtering can effectively distinguish linearly polarized P-wave signals from background noise, and then STA/LTA algorithms can be applied to pick the arrival times^12^. These picking methods have achieved remarkable results in their respective fields, significantly enhancing the performance of arrival time picking.

In the coal mine environment, due to the interference of high-noise signals, existing seismic phase picking technologies still face numerous challenges and limitations in practical applications. Single methods, such as STA/LTA and AIC, struggle to distinguish subtle differences between noise and signals when dealing with low-magnitude or low signal-to-noise ratio (SNR) events, leading to picking errors. Furthermore, digital filters often require specific parameters to be set based on the dominant frequency of the microseismic data, which not only increases operational complexity but may also result in unstable filtering performance and suboptimal outcomes^13^. Current microseismic phase picking models are constrained in coal mine environments by the scarcity of high-quality training data. Most of these models rely on transfer learning from natural earthquake phase picking models. Although improvements in picking accuracy have been observed, their generalization capability is limited when applied to coal mine data under varying geological conditions^10^. Additionally, these models exhibit instability when processing high-noise, non-stationary signals, and in some cases, their performance still falls short of manual picking accuracy. Therefore, there is an urgent need to develop a method that can efficiently and stably extract high-SNR microseismic data in complex and noisy coal mine environments, thereby enhancing the effectiveness and reliability of microseismic monitoring applications.

This paper proposes a microseismic P-wave first arrival picking method, IWTSE-MSDACF-AIC, specifically designed for high-noise coal mine environments. The method consists of two key steps: denoising and automatic picking, aimed at improving the accuracy and stability of signal picking in low SNR conditions. In the denoising process, the Improved complete ensemble empirical mode decomposition with adaptive noise (ICEEMDAN) is employed to decompose the microseismic signal into a series of IMFs. The sample entropy (SamEn) of each IMF is then calculated to identify the primary noise components, which are subsequently processed using wavelet threshold denoising (WTD). After denoising, the sample entropy of the IMFs is recalculated to select the relevant IMFs, and the signals are reconstructed to distinguish between noise and useful signals.

For P-wave first arrival picking, this study introduces an Adaptive Characteristic Function (ACF) based on relative energy coefficients and time series to construct the characteristic function. This is combined with Moving Standard Deviation (MSD) and the Akaike Information Criterion (AIC) to automatically pick the P-wave first arrivals from the denoised microseismic signals. To verify the effectiveness of the proposed method, it was applied to a dataset collected from the KJ874 microseismic network at Dongtan coal mine. The results show that 90.01% of the data have a picking error within the range of 0 to 0.06 s compared to manual picking, demonstrating high accuracy and stability. These findings suggest that the proposed method is well-suited for real-world coal mine environments and holds promising potential for application.

## Method

### Multi-denoising strategy - IWTSE

#### ICEEMDAN

First, we apply the ICEEMDAN decomposition to the seismic records. ICEEMDAN is an advanced version of EMD, EEMD^14^ and CEEMDAN^15^. ICEEMDAN enhances the separation of each IMF, significantly improving the accuracy and stability of signal processing. As a result, ICEEMDAN is capable of delivering more accurate and robust results when handling complex signals, effectively reducing mode mixing and achieving more precise signal decomposition^16^.

Set the noise amplitude parameter and generate a noise sequence$\:\:n\left(i\right)$, adding Gaussian white noise to the original signal to obtain $\:X\left(i\right)$:

1 $$\:X\left(i\right)=X+{\epsilon\:}_{0}{E}_{1}\left(n\left(i\right)\right),\:i=\text{1,2},...,N$$

where $\:X$ is the original signal, $\:{{\upepsilon\:}}_{0}$ is the amplitude coefficient of the added Gaussian white noise, $\:\:{E}_{1}\left(\bullet\:\right)$ denotes the ICEEMDAN operator used to extract the first Intrinsic Mode Function, and $\:i$ represents the signal sample number.

The first residual value is obtained as:

2 $$\:{R}_{1}=\frac{1}{N}\sum\:_{i=1}^{N}M\left(X\left(i\right)\right)$$

where $\:M\left(\bullet\:\right)$ represents the local mean value of the original signal produced by EMD.

Define $\:k$ as the mode index generated by ICEEMDAN decomposition. When $\:k=1$, the first mode $\:{F}_{1}$ is obtained:

3 $$\:{F}_{1}=X-{R}_{1}$$

For $\:k=\text{2,3},4,...,K$, calculate the $\:k-th$ residual $\:{\text{R}}_{\text{k}}$ and the $\:k-th$ mode $\:{F}_{k}$ as follows:

4 $$\:{R}_{k}=\frac{1}{N}\sum\:_{i=1}^{N}M\left({R}_{k-1}+{\epsilon\:}_{k-1}{E}_{k}\left(n\left(i\right)\right)\right)$$

5 $$\:{F}_{k}={R}_{k-1}-{R}_{k}$$

Repeat the calculations to obtain all IMFs until the final residual $\:{R}_{n}$ satisfies the monotonicity condition and can no longer be decomposed. The original signal is then decomposed into two parts:

6 $$\:X={R}_{n}+\sum\:_{i=1}^{N}{F}_{i}$$

**Fig. 1:**
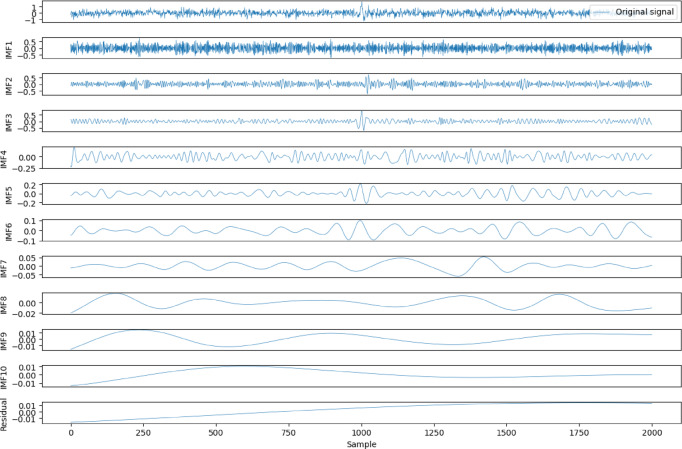
Decomposition of Synthetic Seismic Records Using ICEEMDAN.

In this study, we first validate the proposed method using synthetic seismic records. The synthetic records are sampled at 500 Hz, with a total of 2000 samples. These records were generated by superimposing multiple randomly generated Ricker wavelets, with a dominant frequency range between 30 Hz and 100 Hz. The amplitude of each wavelet was randomly scaled to simulate the complexity of real seismic signals. Gaussian white noise was added to the seismic records to achieve a SNR of −12 dB. Following the recommendations of Wu and Wang^17^ (2009), we selected a noise amplitude such that the added white noise was approximately 20% of the standard deviation of the decomposed data. The signal was then decomposed into 10 IMFs using ICEEMDAN, as shown in Fig. 1. It should be emphasized that the number of IMFs is not predetermined; instead, it is adaptively determined by the ICEEMDAN algorithm itself. The decomposition automatically terminates when the residual becomes a monotonic function that no longer contains oscillatory components, ensuring that the resulting IMFs reflect the intrinsic frequency characteristics of the signal rather than being constrained by a fixed decomposition level or the dominant frequency range.

#### Sample entropy

After obtaining a series of microseismic records using ICEEMDAN, it is essential to identify which components contribute significantly to reconstructing a new microseismic signal. In this study, we use sample entropy (SampEn) to assess the complexity and randomness of the microseismic signals. Specifically, a lower SampEn indicates higher self-similarity and lower noise in the signal, while a higher SampEn corresponds to greater noise and randomness^18,19^.

Let$\:{C}_{k}\left(i\right)$ represent the IMF sequence obtained from ICEEMDAN decomposition, where $\:i=\text{1,2},3,...,N$, and $\:\text{k}=\text{1,2},...,\text{K}$,with $\:\text{K}$ being the total number of IMFs.

For each IMF $\:{C}_{k}\left(i\right)$,construct an m-dimensional vector sequence $\:\text{Y}\left(\text{i}\right)$ as

7 $$\:Y\left(i\right)=\left[{C}_{k}\left(i\right),{C}_{k}\left(i+1\right),...,{C}_{k}\left(i+m-1\right)\right],i=\text{1,2},..,{n}_{m}$$

Where $\:{n}_{m}=N-m+1$ is the number of available vectors of dimension m.

We calculate the distance between sequence $\:\text{Y}\left(\text{i}\right)$ and all sequences $\:\text{Y}\left(\text{j}\right)$, where the distance is defined as:

8 $$\:{d}_{ij}=max\left|{C}_{k}\left(i+l\right)-{C}_{k}\left(j+l\right)\right|$$

$$\:\text{W}\text{h}\text{e}\text{r}\text{e}\:i\ne\:j,\:\text{a}\text{n}\text{d}\:l=\text{0,1},2,...,m-1.$$

Define $\:\text{T}=\text{r}\times\:\text{S}\text{D}$, where r typically ranges between 0.1 and 0.25, and SD represents the standard deviation of the calculated series. Next, count the number of distances $\:{d}_{ij}$ that exceed $\:T$, denoted as $\:{\text{B}}_{\text{i}}^{\text{m}}$. Define $\:{\text{A}}_{\text{i}}^{\text{m}}$, as the ratio of these counts for the $\:i$-th $\:\text{m}$-dimensional sequence, which represents the average similarity of the sequence at the current dimension $\:m$:

9 $$\:{A}_{i}^{m}=\frac{1}{n-m}\times\:{B}_{i}^{m}$$

The average of $\:{A}_{i}^{m}$ is denoted as $\:{\varphi\:}^{m}$, which represents the overall average similarity for dimension $\:m$:

10 $$\:{\varphi\:}^{m}=\frac{1}{n-m}\times\:\sum\:_{i=1}^{n-m}{A}_{i}^{m}$$

Let $\:m=m+1$, and repeat steps(8) to (11).This means recalculating all relevant distances and averages.

SampEn is defined as:

11 $$\:SamEn=ln{\varphi\:}^{m}-ln{\varphi\:}^{m+1}$$

Where $\:{{\upvarphi\:}}^{\text{m}}$ and $\:{{\upvarphi\:}}^{\text{m}+1}$ are the average values calculated for dimensions $\:\text{m}$ and $\:\text{m}+1$.

In this study, $\:m$ and $\:T$ are two key parameters. Based on the research by Zhang et al.^13^$\:m$ is typically set to 1 or 2, and $\:T$ is generally between 0.1 and 0.25 times the standard deviation of the signal. In this study, we set $\:m=1$ and $\:T$ as 0.2s the standard deviation of the IMFs to distinguish the characteristics of different IMFs.

#### Wavelet threshold denoising

The original signal contains high-frequency components, and while the ICEEMDAN algorithm and sample entropy-based denoising effectively reduce noise, they may inadvertently discard some IMF components with significant features, leading to the loss of useful signals. To address this issue, we propose an improvement: we apply WTD to the IMFs exceeding a certain threshold and then recalculate the sample entropy of the denoised IMFs. If the sample entropy exceeds a preset threshold, the component is discarded; otherwise, it is retained.

The advantage of wavelet transform lies in its ability to decompose both high- and low-frequency parts of the signal, providing superior resolution, particularly in handling the high-frequency components associated with sudden signal changes. Therefore, during the secondary processing of IMF components using wavelet threshold denoising, we can effectively eliminate the residual high-frequency components of the white noise while preserving the critical high-frequency features of the signal. This improvement ensures that noise is reduced while maximizing the retention of useful signals^20^.

The core idea of wavelet threshold denoising is to process noisy signals using discrete wavelet transform. Larger wavelet coefficients typically represent useful signal components, whereas smaller coefficients usually contain noise. Thus, thresholds are set at different decomposition levels, specifically targeting the high-frequency wavelet coefficients to reduce noise. Subsequently, the signal is reconstructed through inverse wavelet transform, ultimately yielding the denoised signal. The calculation formula for discrete wavelet transform is as follows:

12 $$\:WT\left(a,b\right)=\frac{1}{\sqrt{{2}^{a}}}\sum\:_{t=1}^{T}s\left(t\right)\times\:\psi\:\left(\frac{t-b\bullet\:{2}^{a}}{{2}^{a}}\right)$$

Where $\:\text{a}$ is the decomposition level, b is the translation parameter, $\:\psi\:$ is the wavelet basis function, and $\:T$ is the sampled data.

The key elements of wavelet threshold denoising include the selection of the wavelet basis, the number of decomposition levels, and the application of the threshold function, all of which directly impact the denoising performance. The denoising process typically involves the following steps:

1. Select an appropriate wavelet basis function and decomposition level for discrete wavelet decomposition;
2. Set the threshold value λ, and choose the corresponding threshold function to apply to the wavelet coefficients at each decomposition level;
3. Reconstruct the signal to obtain the denoised result.

When selecting a wavelet basis, it is important to consider the characteristics of the signal and the mathematical properties of the wavelet basis function. Common wavelet basis functions include Haar, DB, and sym functions. In this study, the DB wavelet was chosen as the wavelet basis. Its advantages lie in its high compact support and excellent frequency resolution, making it effective in capturing abrupt features in microseismic signals. Although the DB wavelet may cause slight phase distortion in some cases, it demonstrates good time-frequency localization properties when handling complex non-stationary signals, making it suitable for the data environment in this study.

Regarding the choice of decomposition levels, more levels help to better separate the signal from the noise, but they may introduce reconstruction distortion and reduce the SNR. For non-smooth signals, at least two levels of decomposition are necessary. Based on multiple tests, we selected four levels of decomposition, which achieves a balance between signal reconstruction and noise suppression.

Threshold selection is critical, as an overly large or small threshold λ can affect the denoising results. Once an appropriate threshold function is selected, different methods are applied to wavelet coefficients depending on whether they exceed the threshold λ. Generally, threshold functions are divided into hard threshold functions and soft threshold functions, defined as follows:

Hard Threshold Function:

13 $$\:{\widehat{\text{w}}}_{\text{j},\text{k}}=\left\{\begin{array}{c}{\text{w}}_{\text{j},\text{k}},\:\:\left|{\text{w}}_{\text{j},\text{k}}\right|\ge\:\lambda\:\\\:0,\:\left|{\text{w}}_{\text{j},\text{k}}\right|\le\:\lambda\:\end{array}\right.$$

Soft Threshold Function:

14 $$\:{\widehat{w}}_{j,k}=\left\{\begin{array}{c}sgn\left({w}_{j,k}\right)\left(\left|{w}_{j,k}\right|-\lambda\:\right),\:\:\left|{w}_{j,k}\right|\ge\:\lambda\:\\\:0,\:\left|{w}_{j,k}\right|\le\:\lambda\:\end{array}\right.$$

Where $\:{w}_{j,k}$ and $\:{\widehat{w}}_{j,k}$ are the wavelet coefficients before and after processing, respectively, and $\:sgn\left(\bullet\:\right)$ is the sign function.

The hard threshold function consists of a step-like structure, which can introduce oscillations at the edges of the denoised signal. In contrast, the soft threshold function compensates for the discontinuity of the hard threshold function, resulting in a relatively smoother denoised signal waveform. In practice, both have their limitations. However, in most cases, the soft threshold function performs better than the hard threshold function. Therefore, in this study, we adopt the soft threshold function for denoising.

**Fig. 2:**
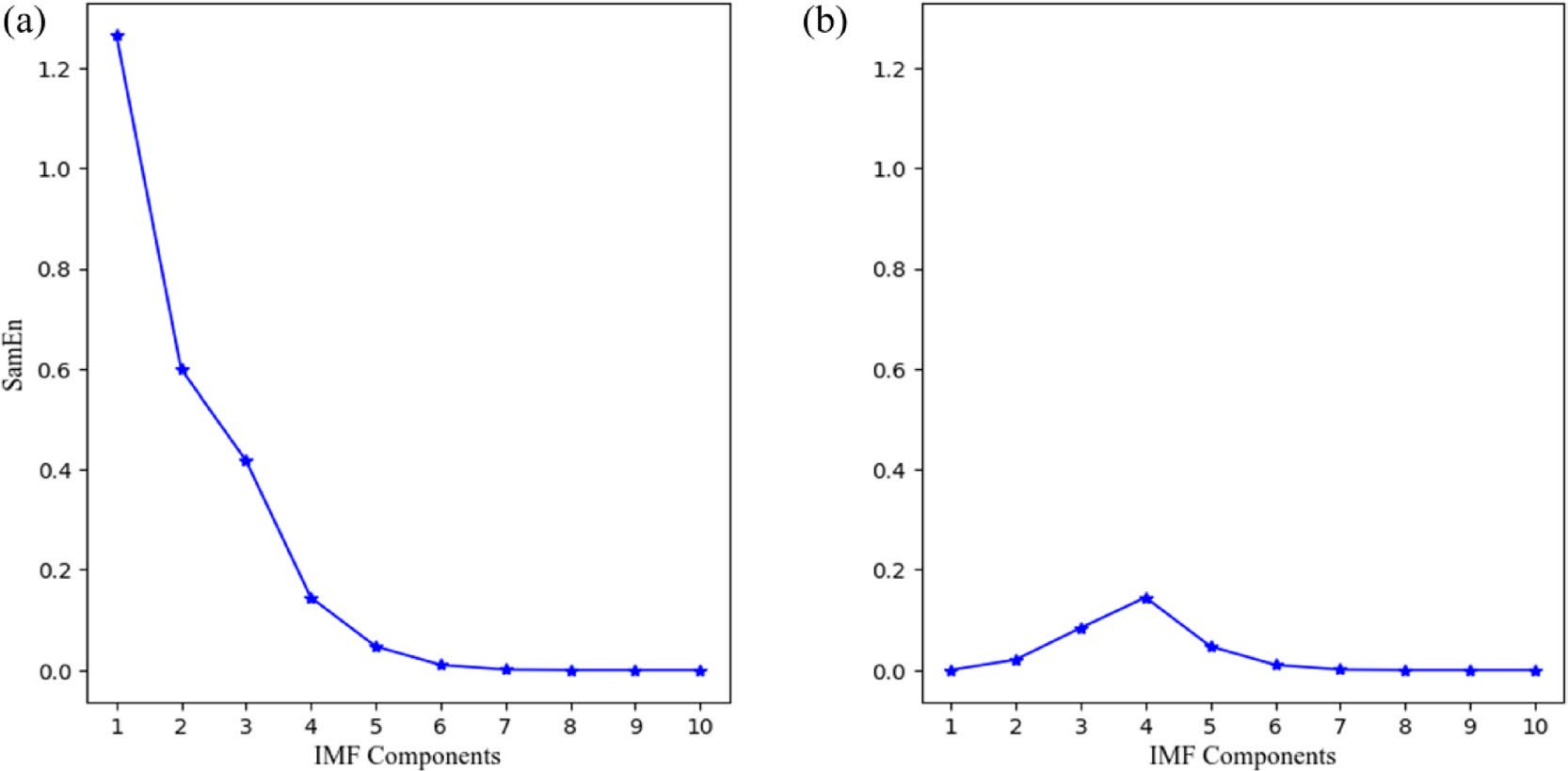
Sample entropy changes of IMF components. (**a**) Sample entropy of IMF components; (**b**) Changes in sample entropy after WTD denoising.

**Fig. 3:**
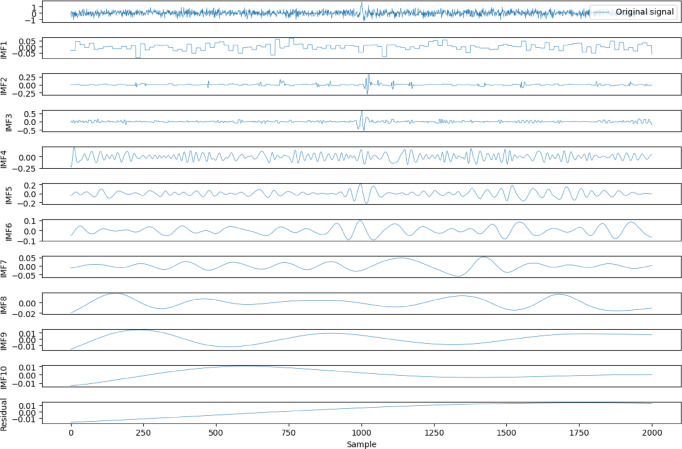
IMF components after WTD denoising.

To distinguish the different characteristics of the IMFs, we calculated the sample entropy of the IMF components shown in Fig. 1, as illustrated in Fig. 2(a). To extract the useful IMF components, a reasonable threshold needs to be determined to differentiate the noise-containing IMFs. By assessing the noise characteristics of the signal through randomness^13^ we set the threshold at 0.2. During the tests, the sample entropy of IMF1, IMF2, and IMF3 exceeded this threshold, so we applied wavelet threshold denoising to these components. The denoised IMF components are shown in Fig. 3. After denoising, the sample entropy was recalculated, as shown in Fig. 2(b), where the sample entropy of all IMFs was below 0.2. These IMFs were then combined to obtain the reconstructed data.

**Fig. 4:**
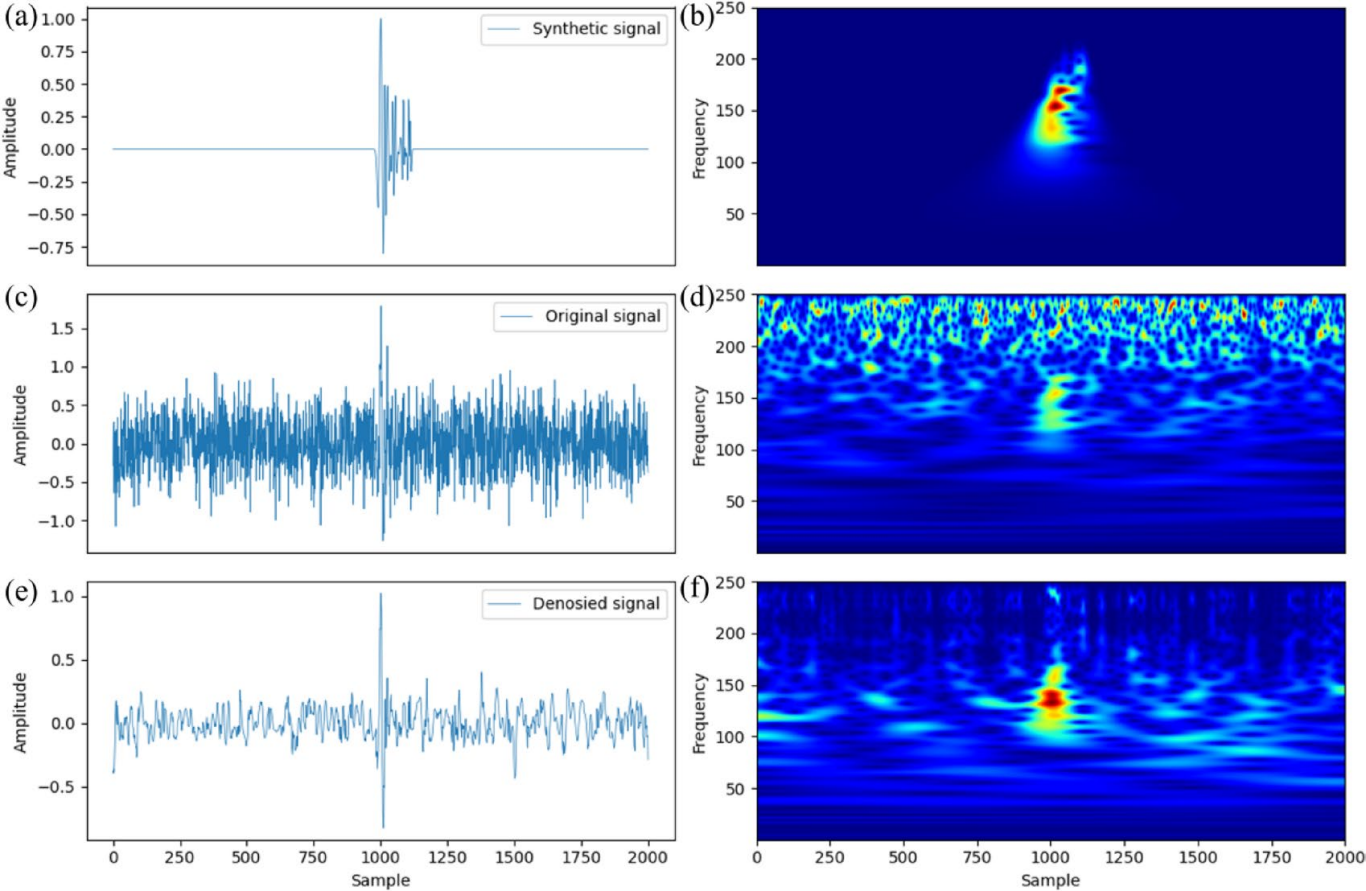
Theoretical synthetic seismic record (**a**), synthetic seismic record with added Gaussian noise (−12 dB) (**c**), IWTSE-denoised and reconstructed seismic record (**e**), and their corresponding time-frequency diagrams(**b**,**d**,**f**).

We named this multi-stage denoising strategy IWTSE. The microseismic records denoised using this method were then used as input for the new picking method. To evaluate the ability of IWTSE to reconstruct the signal, we performed CWT time-frequency analysis on three datasets: the synthetic seismic record, the noisy record with added Gaussian noise, and the signal reconstructed using IWTSE. The results are presented in Fig. 4. Figure 4(a) shows the synthetic seismic record without noise, and Fig. 4(e) presents the denoised record obtained by applying IWTSE to Fig. 4(c). The denoised synthetic seismic record achieved an SNR of −2.91 dB and a root mean square error of 0.11, indicating that the denoised signal closely resembles the original. Figure 4(b) provides the time-frequency representation of the synthetic waveform. Comparing Figs. 4(d) and 4(f), we observe that the energy distribution in Fig. 4(f) is more concentrated, noise is effectively suppressed, and the spectral characteristics are more consistent with the synthetic seismic record. These results demonstrate that the proposed denoising strategy preserves the main features of the original record and achieves high signal fidelity. Therefore, the denoised signal is suitable for reliable P-wave arrival time picking.

### P-wave onset picking method

The arrival time of the P-wave in a mining-induced seismic event can be determined by amplifying and analyzing the changes in amplitude and frequency within the seismic sequence. The primary task of the characteristic function is to capture these changes, thereby enhancing the ability to distinguish between signal and noise. By constructing a reasonable and sensitive characteristic function, the resolution between signal and noise can be further improved, thereby enhancing the accuracy of P-wave arrival time determination^21–23^. Absolute value characteristic functions and squared characteristic functions are among the earliest and most widely applied characteristic functions; however, these functions only indicate changes in amplitude and are only applicable to signals with high SNR.

In this study, considering the weaker suppression of weak amplitudes by the squared characteristic function, we introduce a relative time series and relative energy coefficient to establish an adaptive characteristic function.Suppose $\:x\left(i\right)$ represents the amplitude sequence of a seismic event, and $\:W$ is a weight parameter that changes with the sampling rate and stationary noise characteristics, expressed as:

15 $$\:W\left(i\right)=\sum\:_{j=1}^{i}{x}^{2}\left(j\right)/\sum\:_{j=1}^{i}{\left[x\left(j\right)-x\left(j-1\right)\right]}^{2}$$

$\:{X}_{max}$ represents the maximum absolute amplitude of $\:x\left(i\right)$, used to adjust the time series in the seismic record, and the relative time series $\:\text{X}\left(\text{i}\right)$ is as follows:

16 $$\:X\left(i\right)=\left|x\left(i\right)/{X}_{max}\right|$$

The relative energy coefficient $\:{\upalpha\:}$ is used to differentiate between noise and the signal-dominant regions, and it is defined as:

17 $$\:\alpha\:=2{e}^{-\frac{{W}^{2}}{2}X\left(i\right)}$$

The ACF is modified from the characteristic function proposed by Allen (1982) and is expressed as:

18 $$\:ACF\left(i\right)={x}^{\alpha\:}\left(i\right)+W\bullet\:{\left[x\left(i\right)-x\left(i-1\right)\right]}^{2}$$

**Fig. 5:**
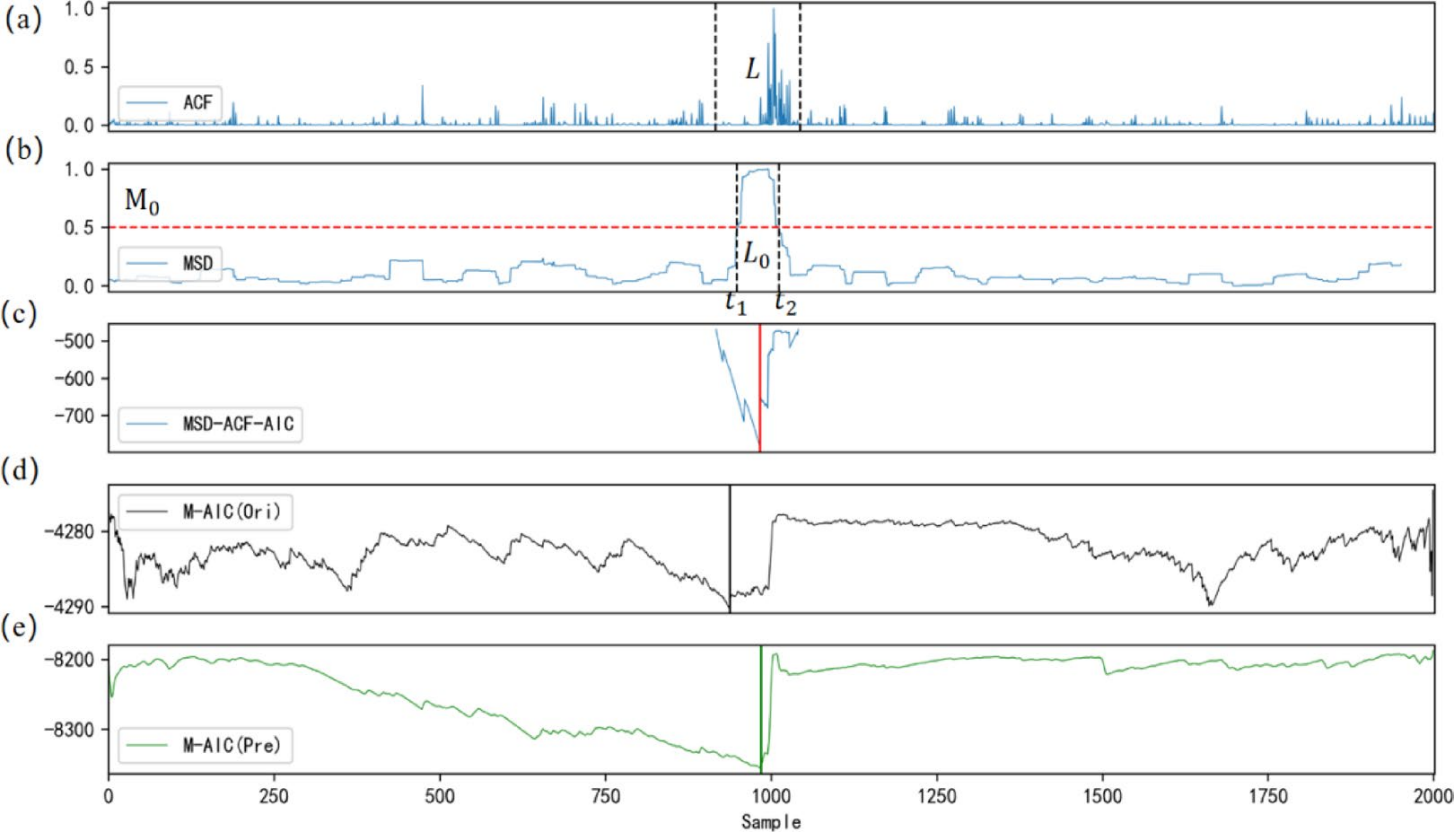
Flowchart of the IWTSE-MSDACF-AIC picking method and comparison of results before and after denoising with the M-AIC method.

By calculating each characteristic value based on Eq. (18), we normalize the results to obtain an adaptive characteristic function, as shown in Fig. 5(c), where the ACF can further suppress effective noise data.

To further improve the accuracy of P-wave onset picking, we integrate the ACF with the MSD and M-AIC methods. First, the MSD is calculated for the ACF, and two fundamental parameters are set: sliding window $\:{\Delta\:}{\text{T}}_{0}$ and threshold $\:{M}_{0}$. In this study, the sliding window is set to 0.15s, and the picking threshold $\:{M}_{0}$ is set to 0.5.The MSD of the ACF is calculated as follows:

19 $$\:M\left(i\right)=\sqrt{\frac{1}{N}\sum\:_{j=i}^{i+N-1}{\left(ACF\left(j\right)-{\mu\:}_{i}\right)}^{2}}$$

Where $\:{\mu\:}_{i}$ is the moving average of the sliding window, expressed as:

20 $$\:{\mu\:}_{i}=\frac{1}{N}\sum\:_{j=i}^{i+N-1}ACF\left(j\right)$$

Using the calculated $\:M\left(i\right)$ and threshold $\:{\text{M}}_{0}$,we determine the basic time interval $\:{L}_{0}$, as shown in Fig. 5(d).The length between the two black dashed lines represents the basic time interval $\:{L}_{0}$, and the two points where $\:{M}_{i}$ crosses correspond to times $\:{t}_{1}$ and $\:{t}_{2}$, respectively.The maximum value of $\:{M}_{i}$ falls between these two points.

In the second step, based on the $\:{t}_{1}$ and $\:{t}_{1}$ determined by the MSD and the basic time interval $\:{\text{L}}_{0}$,we determine the length $\:\text{L}$ of the AIC window. The length of AIC window should be sufficient to encompass the prominent waveform.In this study, the AIC window length is controlled by $\:N\times\:{L}_{0}$, Where $\:\text{N}$ is adjusted between 2 and 4. In this paper, $\:\text{N}$ is set to 2, and the AIC window is determined ad the distance between the two black dashed lines in Fig. 5(a).

We then apply the M-AIC to refine the ACF-based detection. M-AIC is an enhanced version of the traditional AIC that improves robustness under noisy conditions by incorporating the signal’s structural characteristics. The ACF variance is used to replace the time series variance, as shown in Eq. (21):

21 $$\:\text{A}\text{I}\text{C}\left(\text{i}\right)=\left(\text{i}-2\right)\text{l}\text{o}\text{g}\left(\text{v}\text{a}\text{r}\left(\text{A}\text{C}\text{F}\left[1,\text{i}\right]\right)\right)+(\text{L}-2-\text{i})\text{l}\text{o}\text{g}\left(\text{v}\text{a}\text{r}\left(\text{A}\text{C}\text{F}\left[\text{i}+1,\text{L}\right]\right)\right)$$

Where $\:\text{L}$ is the length of the AIC window, and $\:\text{v}\text{a}\text{r}\left(ACF\left[1,i\right]\right)$ is the variance of the ACF from 1 to $\:i$, where $\:i=\text{1,2},..,L$.

After completing the above steps, the ACF of the determined window is calculated using the AIC method. By searching for the minimum value of the local AIC window, the P-wave arrival time is finally determined. As shown by the red solid line in Fig. 5(c), the minimum value of the AIC corresponds to the P-wave arrival time in the original microseismic record, with a P-wave index value of 985. The theoretical synthetic seismic record has a P-wave index value of 986, resulting in an error of 0.002s.

**Fig. 6:**
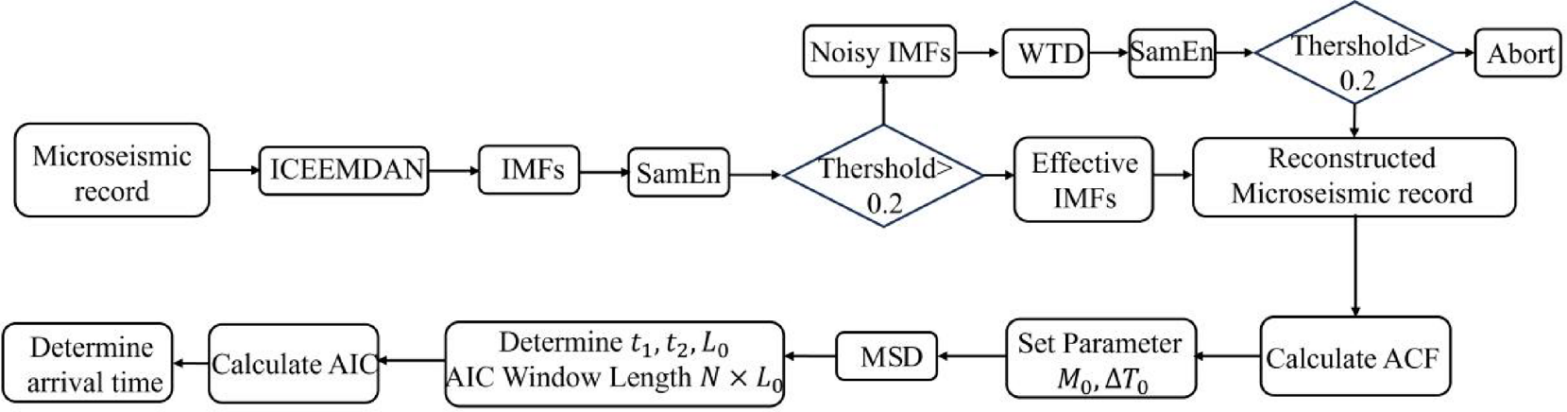
Flowchart of the IWTSE-MSDACF-AIC P-wave onset picking process.

**Fig. 7:**
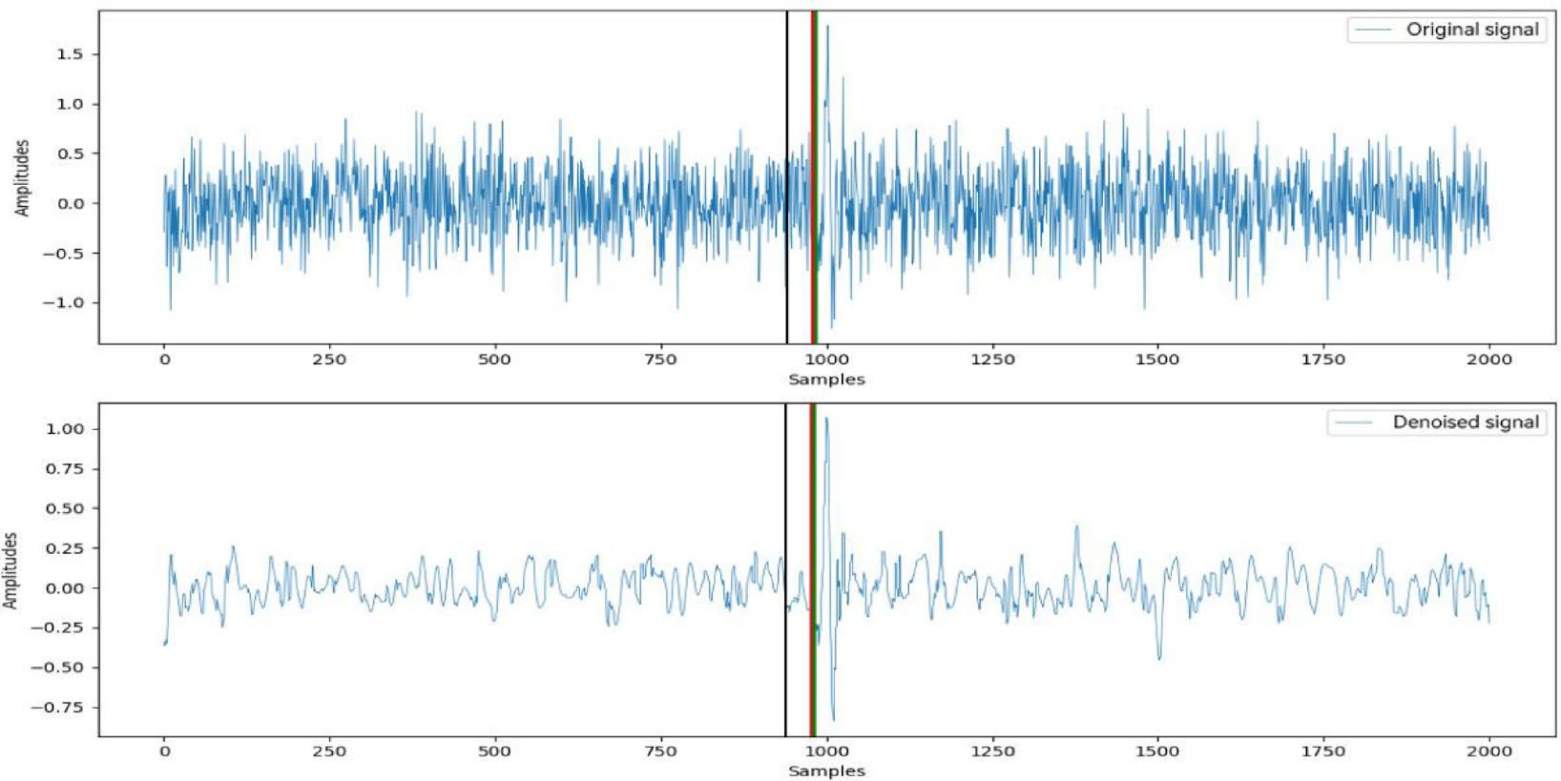
Comparison of Picking Results.

Figure 6 presents the overall picking flow of the IWTSE-MSDACF-AIC method, encompassing key steps such as denoising, feature extraction, and P-wave onset identification. Figure 5 illustrates the step-by-step application of the MSDACF-AIC picking method to a real microseismic waveform and provides a comparative analysis of picking results before and after denoising using the M-AIC method. Specifically, Fig. 5(a) shows the ACF of the reconstructed signal, Fig. 5(b) displays the calculated MSD, and Fig. 5(c) presents the MSDACF-AIC function derived from the MSD and ACF, where the minimum value corresponds to the P-wave onset. Figures 5(d) and 6(e) depict the M-AIC picking results before and after denoising, with picking indices of 938 and 984, and corresponding errors of 0.096 s and 0.004 s, respectively. Figure 7 further compares the picking results of the three methods in the time domain. The upper panel shows the original signal, while the lower panel displays the denoised signal obtained via the IWTSE method. In these plots, the red solid line represents the P-wave onset identified by the proposed IWTSE-MSDACF-AIC method, whereas the black and green solid lines correspond to the M-AIC results before and after denoising, respectively. The comparison clearly demonstrates that the IWTSE denoising strategy significantly improves signal quality and enhances the accuracy of onset detection, verifying the effectiveness and robustness of the proposed method in microseismic P-wave picking.

## Result and discussion

### Synthetic record testing and comparison

To evaluate the effectiveness of the proposed method and the influence of different SNR levels on picking performance, this study compares the proposed approach with traditional STA/LTA and M-AIC picking methods, as well as wavelet transform -based denoising methods. The parameters for STA/LTA were set according to the standards proposed by Akram and Eaton^12^. In the experiments, Gaussian noise at different SNR levels was added to synthetic microseismic records, with SNRs ranging from − 10 dB to 10 dB in 2 dB increments, resulting in 11 different SNR levels. For each SNR condition, 50 synthetic microseismic records were randomly generated. The P-wave arrival times were picked using different methods, and the average picking accuracy was evaluated.

**Fig. 8:**
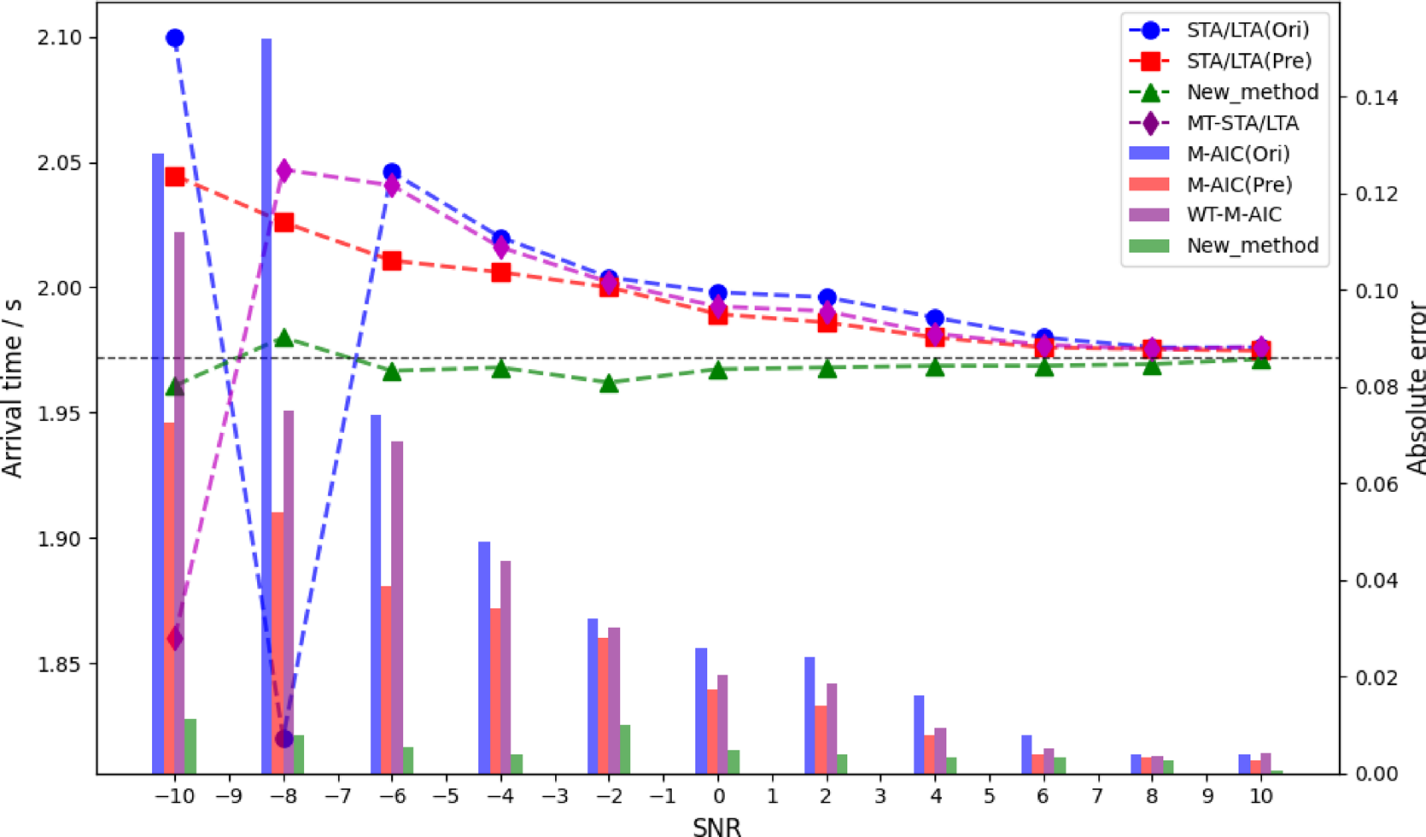
Comparison of IWTSE-MSDACF-AIC method and STA/LTA picking methods under different SNR conditions.

**Fig. 9:**
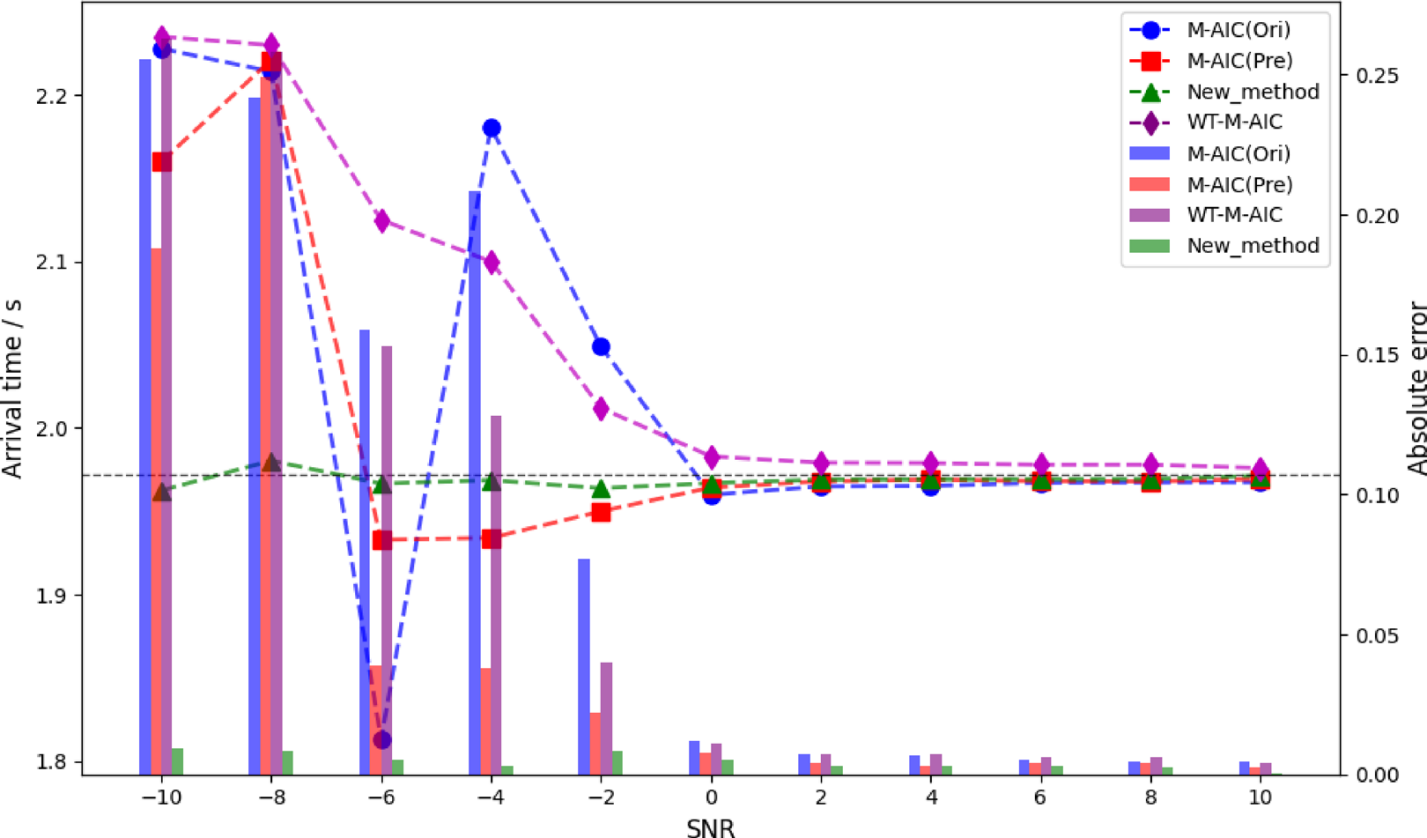
Comparison of IWTSE-MSDACF-AIC method and M-AIC picking methods under different SNR conditions.

Figures 8 and 9 show the picking results of the M-AIC and STA/LTA methods under different SNR conditions. “Ori” represents the original data, “Pre” represents the data denoised by IWTSE, “New_method” refers to the proposed method, and “WT-M-AIC” and “WT-STA/LTA” indicate the methods using wavelet transform for denoising. The dots in the line plots indicate the picked P-wave arrival times, and the bar charts show the absolute errors. The theoretical P-wave arrival time is 1.972 s. The experimental results show that under low SNR conditions (−10 dB to −6 dB), the traditional STA/LTA and M-AIC methods perform unstably on the original data, with large errors and significant fluctuations. After IWTSE denoising, the performance of these traditional methods is significantly improved. For example, the picking error of M-AIC(Pre) at −10 dB is reduced from 0.26 s to 0.18 s, and STA/LTA(Pre) shows a similar improvement. In contrast, although the wavelet transform methods show some improvement under low SNR, their improvement is significantly less than that of IWTSE, and large errors and instability still occur under − 10 dB and − 8 dB. At moderate SNR levels (−4 dB to 0 dB), the advantage of IWTSE becomes more evident. The picking accuracy of M-AIC(Pre) and STA/LTA(Pre) is significantly better than that of the corresponding wavelet-based methods. This indicates that IWTSE has a stronger denoising capability than traditional wavelet transform, effectively preserving useful signal information while suppressing noise. As SNR increases further (2 dB to 10 dB), the performance differences among the methods gradually decrease, but the proposed method still maintains the best picking accuracy. Under high SNR conditions, the performance of the traditional methods with IWTSE denoising approaches that of the proposed method, while the improvement of the original and wavelet-based methods remains limited.

The comparative analysis shows that IWTSE denoising has significant advantages over traditional wavelet transform methods in signal preprocessing, as it better preserves signal features and suppresses noise interference. The proposed IWTSE-MSDACF-AIC method demonstrates excellent picking performance across all SNR levels, with significantly lower picking errors than traditional methods under low SNR conditions, showing superior noise resistance and robustness.

### Case study

To verify the practical application of the proposed picking method, we selected a microseismic dataset from the Dongtan coal mine for testing. This dataset contains 343 microseismic records, each with manually annotated P-wave arrival times. The data has been carefully corrected, making it suitable for accuracy comparison with the automatic picking methods.

**Fig. 10:**
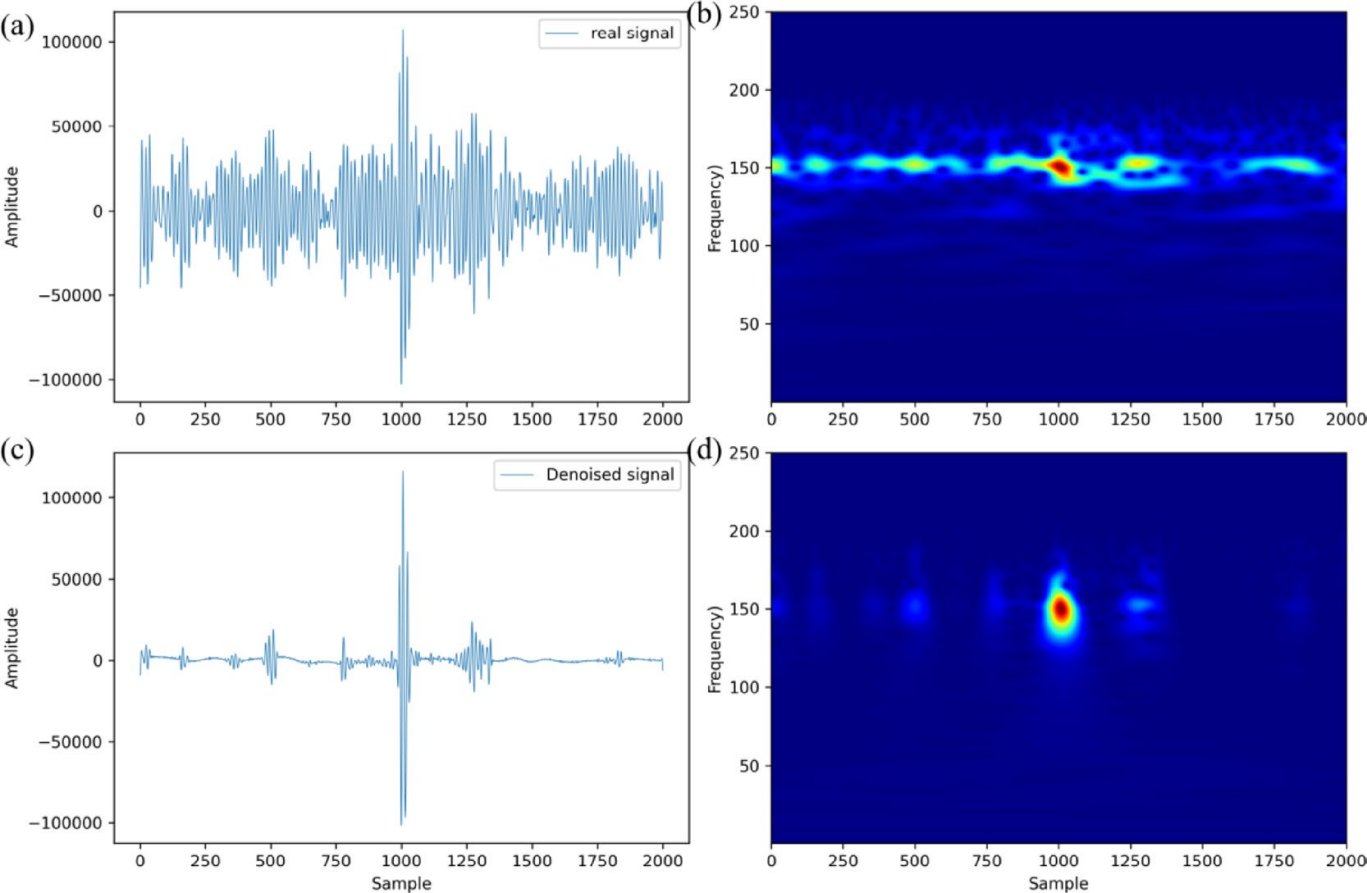
Waveforms and time-frequency diagrams of the actual microseismic data before (**a**, **b**) and after (**c**, **d**) denoising.

As an example, Fig. 10 illustrates the denoising process of a microseismic event recorded by station N1. Figure 10(a) presents the original Z-component waveform, which contains a significant amount of high-frequency noise. The corresponding time-frequency representation in Fig. 9(b) reveals a persistent high-frequency noise band across the entire time window, severely affecting the identification and analysis of the microseismic signal. After applying the IWTSE denoising algorithm, the waveform shown in Fig. 10(c) becomes significantly clearer, with a substantial reduction in noise components. The corresponding time-frequency plot in Fig. 10(d) confirms that the high-frequency noise band has been effectively suppressed, and the signal’s frequency components appear more concentrated and distinct. The SNR improves from 6.4 dB to 15.6 dB, demonstrating that this denoising technique can significantly enhance signal quality and improve the reliability of microseismic signal analysis.

**Fig. 11:**
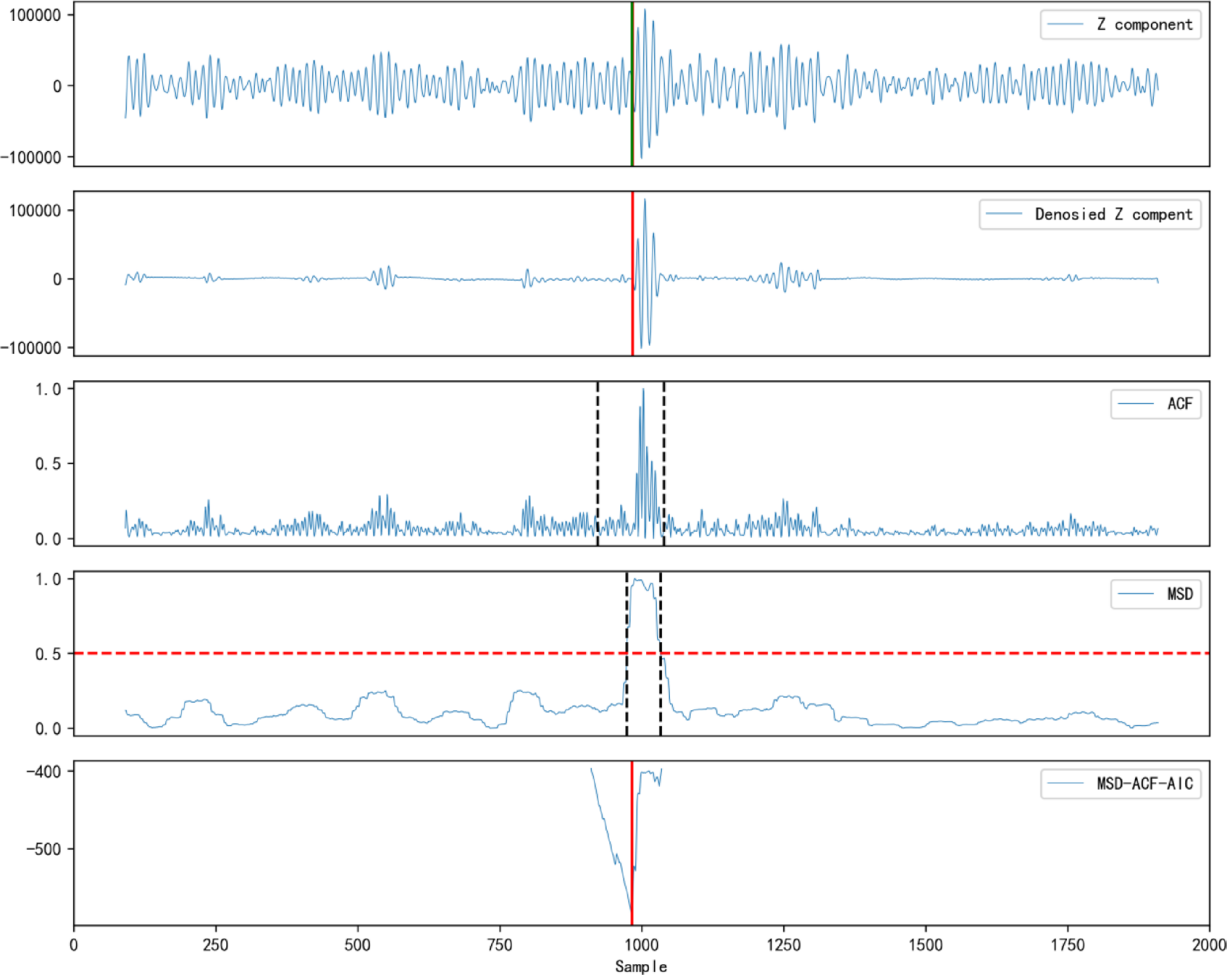
Comparison of manual picking and IWTSE-MSDACF-AIC method picking results for actual microseismic records.

Figure 11 presents the overall workflow of the IWTSE-MSDACF-AIC method for P-wave arrival picking. Figure 11(a) shows the original waveform, while Fig. 11(b) displays the waveform after denoising using the IWTSE approach, in which background noise is effectively suppressed. The green solid line indicates the manually annotated P-wave arrival time, whereas the red solid line represents the arrival time identified by the proposed method. Figure 11(c) illustrates the characteristic curve extracted using ACF, with two black dashed lines marking the final search window. Figure 11(d) shows the MSD detector, where the red dashed line denotes the decision threshold, and the two black dashed lines indicate the initial candidate window. Figure 11(e) presents the final decision curve constructed by integrating the ACF and MSD results with the AIC, where the minimum of the curve corresponds to the final identified P-wave arrival time.As shown in the figure, the identified arrival time is highly consistent with the Manual annotation, with a time deviation of only 0.002 s, demonstrating the high accuracy and robustness of the proposed method for microseismic P-wave onset detection.

**Fig. 12:**
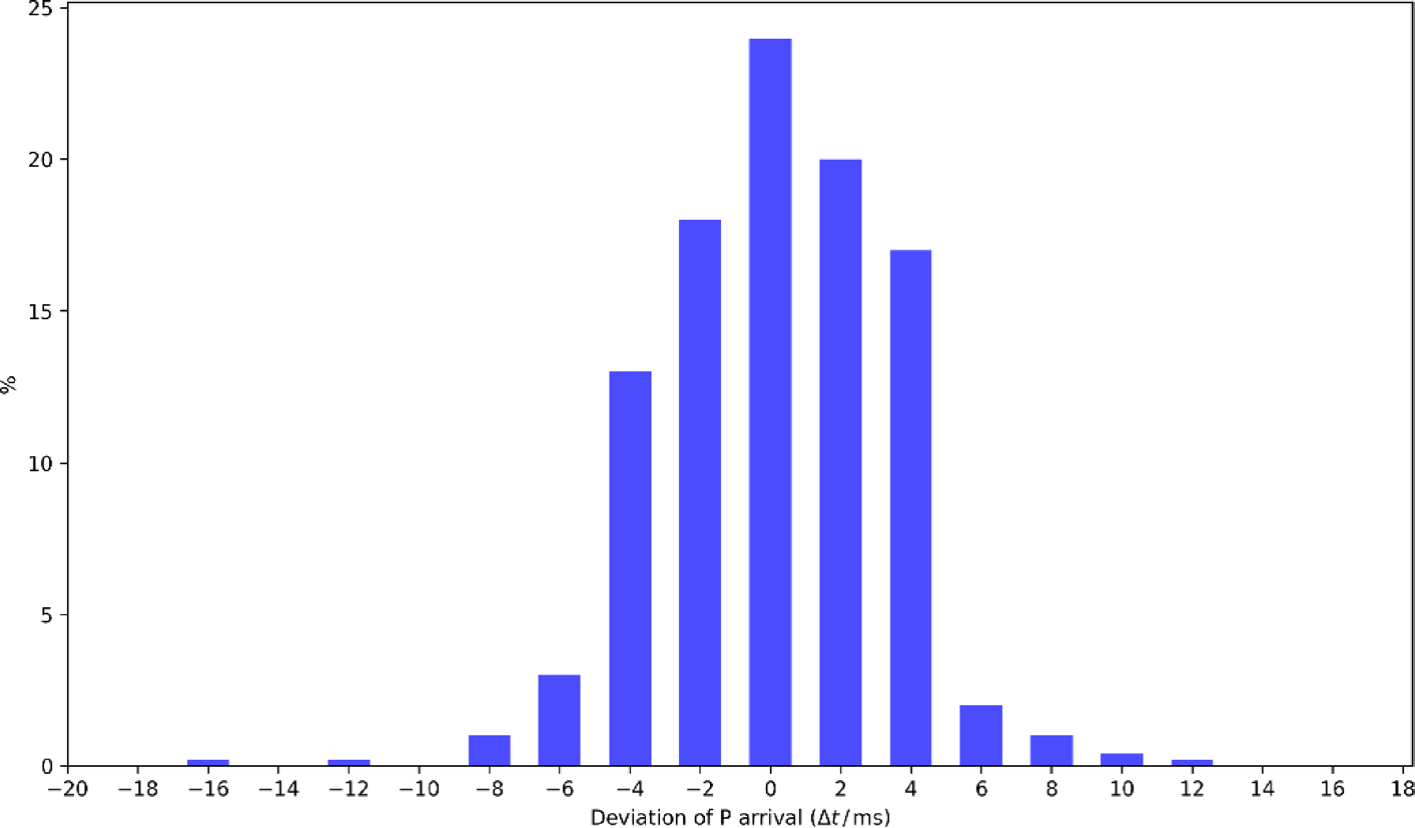
Error deviation histogram comparing manual picking and IWTSE-MSDACF-AIC picking results for Dongtan coal mine.

To further verify the method’s general applicability, we tested the entire dataset of 343 microseismic records. The results show that in 309 records (approximately 90.01%), the automatic picking results had an error within 0 to 3 sample intervals compared to the manual annotations, corresponding to an error range of ± 0ms to ± 6ms. Figure 12 displays the statistical distribution of P-wave arrival time errors, with most errors concentrated around 0ms, exhibiting a typical normal distribution. This result indicates that the automatic picking method has achieved an accuracy comparable to manual picking and demonstrates high stability and consistency across a large dataset.

### Orthogonal testing

For the ACF-based MSD-AIC picker, multiple parameters may affect the picking accuracy, such as the length of the moving standard deviation window, the picking threshold, and the AIC window constant. Additionally, variations in the SNR significantly influence the accuracy of the picking results. To better analyze the effect of these parameters on the algorithm’s performance and to optimize the ACF, we designed a series of experiments based on orthogonal testing. The experiments utilized real coal mine microseismic signals and reconstructed signals at different noise levels for testing.

In the experiments, the arrival time for all signals was set to the same value of 1.94 s to better detect the influence of noise on the picking results. We selected four main parameters: the length of the moving standard deviation window($\:{\Delta\:}{\text{T}}_{0}$), the picking threshold($\:{\text{M}}_{0}$), the AIC window constant (N), and SNR, with each parameter set to five different levels. By designing 25 groups of orthogonal tests, we assessed the sensitivity of each parameter on the localization algorithm, thereby highlighting the optimization potential of the ACF algorithm. The test results are shown in Table 1.

**Table 1:** Orthogonal test table for different parameters of the ACF-based MSD-AIC picking method.

Number	$\:\varDelta\:{\varvec{T}}_{0}$(s)	$\:{\varvec{M}}_{0}$	$\:\varvec{N}$	$\:\varvec{S}\varvec{N}\varvec{R}$	Arrival time(s)
1	0.05	0.2	2	5	1.95
2	0.1	0.2	2.5	5	1.95
3	0.15	0.2	3	5	1.95
4	0.2	0.2	3.5	5	1.936
5	0.25	0.2	4	5	1.932
6	0.05	0.3	2	10	1.932
7	0.1	0.3	2.5	10	1.932
8	0.15	0.3	3	10	1.924
9	0.2	0.3	3.5	10	1.932
10	0.25	0.3	4	10	1.932
11	0.05	0.4	2	15	1.94
12	0.1	0.4	2.5	15	1.94
13	0.15	0.4	3	15	1.932
14	0.2	0.4	3.5	15	1.932
15	0.25	0.4	4	15	1.932
16	0.05	0.5	2	20	1.944
17	0.1	0.5	2.5	20	1.944
18	0.15	0.5	3	20	1.94
19	0.2	0.5	3.5	20	1.94
20	0.25	0.5	4	20	1.93
21	0.05	0.6	2	25	1.94
22	0.1	0.6	2.5	25	1.94
23	0.15	0.6	3	25	1.944
24	0.2	0.6	3.5	25	1.944
25	0.25	0.6	4	25	1.944

From the analysis of the orthogonal experiment results, it can be concluded that the accuracy of the picker is primarily influenced by the SNR and the length of the moving standard deviation window $\:\varDelta\:{T}_{0}$. A higher SNR significantly improves the picking accuracy, while a moderate window length setting (e.g., 0.15 to 0.2 s) proves to be more stable. In contrast, the AIC window constant (N) and the picking threshold ($\:{M}_{0}$) had a smaller impact on the picking results within the tested range. Therefore, in practical applications, enhancing the SNR and selecting an appropriate window $\:\varDelta\:{T}_{0}$ are key to optimizing picker performance.

### Comparative analysis of localization results

To systematically evaluate the localization performance of different P-wave onset picking methods in practical mining applications, this study conducted comparative experiments on five calibration shots from the Dongtan coal mine using station data from the KJ874 seismic network. A grid-search localization algorithm was employed, and all localization parameters were kept identical across methods to ensure comparability. Specifically, the search volume was centered on the known shot coordinates and defined as ± 200 m in the X and Y directions and ± 150 m in the Z direction. A uniform velocity model of 3550 m/s was adopted, and the grid spacing was fixed at 5 m. Since the shot locations were precisely known, the only difference among methods lay in the picked arrival times, such that the resulting localization errors directly reflect the picking accuracy. In the experimental design, we compared four representative picking methods: the classical STA/LTA, the kurtosis detector, the deep-learning-based PhaseNet^24^and the proposed IWTSE–MSDACF–AIC. For each calibration shot, the localization errors in the X, Y, and Z directions, as well as the total error, were calculated to comprehensively assess the accuracy and stability of different methods under complex noise conditions.

The results (Table 2) show that the proposed IWTSE–MSDACF–AIC method consistently achieves substantially lower localization errors than the other methods. For example, in the first calibration shot, the proposed method produced X, Y, and Z errors of 10.12 m, 8.24 m, and 10 m, respectively, yielding a total error of 16.44 m. By contrast, PhaseNet produced errors of 14.52 m, − 23.60 m, and − 25 m (total error 37.32 m), while STA/LTA and kurtosis exhibited even larger deviations, with total errors exceeding 50 m in some cases. In the fourth calibration shot, representing a more complex noise environment, the proposed method limited the Z-direction error to 20 m, markedly outperforming the other methods and highlighting its robustness under challenging conditions.

Experimental results based on KJ874 seismic network data demonstrate that, under identical localization parameters, different picking methods exhibit significant differences in performance. The classical STA/LTA and kurtosis methods produce large and unstable localization errors under noisy conditions; PhaseNet performs better than traditional methods but remains sensitive in low-SNR environments. In contrast, the proposed IWTSE–MSDACF–AIC method consistently achieves smaller errors in the X, Y, and Z directions as well as in the total localization error across all calibration shots, demonstrating higher accuracy and robustness. These findings confirm that, when localization parameters are fixed, picking accuracy is the key factor determining final localization performance. The results not only validate the scientific effectiveness of the proposed method but also highlight its practical potential for reliable microseismic event localization in complex mining environments.

**Table 2:** Localization errors using grid search with different Phase-Picking method.

Method	Event No.	X Error (m)	Y Error (m)	Z Error (m)	Total Error (m)
STA/LTA	1	14.4	−7.89	10	19.24
	2	5.96	−11.97	−6	14.24
	3	18.2	6.58	28	34.05
	4	10.3	−46.69	−36	59.56
	5	29.14	12.31	−48	57.48
Kurtosis	1	25.52	−10.08	−30	38.84
	2	14.28	−11.30	−15	23.59
	3	30.40	−23.65	−32	50.07
	4	36.14	22.20	25	49.23
	5	−15.58	−54.58	−10	57.70
PhaseNet	1	14.52	−23.60	−25.00	37.32
	2	10.26	−5.54	−15	18.89
	3	13.86	12.68	20	27.44
	4	−26.45	−30.21	25	47.29
	5	22.82	16.42	−30	41.11
IWTSE- MSDACF- AIC	1	10.12	8.24	10	16.44
	2	6.43	10.24	5	13.08
	3	−12.48	6.24	15	20.48
	4	12.63	10.98	20	26.08
	5	−9.54	14.54	−10	20.06

## Conclusions

This study presents the IWTSE-MSDACF-AIC method, which integrates multi-level denoising and adaptive feature extraction strategies to provide a high-precision and robust solution for P-wave first arrival picking in coal mine microseismic monitoring under high-noise environments. Experimental results demonstrate that the proposed method exhibits excellent robustness across different SNR conditions, particularly showing significant advantages over traditional methods in low-SNR environments. In practical coal mine microseismic data testing, 90.1% of samples achieved picking errors within ± 0.06s, and the method demonstrated superior performance in three-dimensional seismic event localization accuracy compared to benchmark methods, indicating strong potential for engineering applications. Furthermore, sensitivity analysis of key parameters including SNR and time window length was conducted through orthogonal experiments, providing theoretical foundations for parameter optimization.

However, the proposed method has certain limitations. The multi-level decomposition of ICEEMDAN and sample entropy calculation increase the computational burden, potentially affecting response efficiency in real-time monitoring applications. Additionally, the method involves multiple parameters that require adjustment according to specific geological conditions, increasing deployment complexity. Future research will focus on algorithm simplification, adaptive parameter adjustment, and adaptability to complex signal environments, while conducting validation across diverse geological and monitoring scenarios to enhance its generalizability and engineering application value.

## Data Availability

The datasets used and/or analysed during the current study available from the corresponding author on reasonable request.
